# Study on the Influence of Bio-Based Packaging System on Sodium Benzoate Release Kinetics

**DOI:** 10.3390/foods9081010

**Published:** 2020-07-27

**Authors:** Amalia Conte, Lucia Lecce, Mariapia Iannetti, Matteo Alessandro Del Nobile

**Affiliations:** Department of Agricultural Sciences, Food and Environment, University of Foggia, via Napoli, 25-71121 Foggia, Italy; amalia.conte@unifg.it (A.C.); lucia.lecce@unifg.it (L.L.); mariapia.iannetti@tiscali.it (M.I.)

**Keywords:** controlled release, alginate beads, multilayer, active packaging, chitosan film

## Abstract

The influence of film structure on the release kinetics of sodium benzoate (SB) from polymeric films is addressed in this study. In particular, four film structures were investigated, two monolayer and two multilayer systems. In particular, in one case, the active substance was uniformly distributed into a chitosan-based matrix, and in the other one, it was previously incorporated into alginate beads before dispersion in the chitosan film, thus realizing two types of monolayer films; on the other hand, the same chitosan film with SB encapsulated in alginate beads was used as the inner layer of a multilayer system constituted by two side films of alginate. The two alginate-based layers were made with two different thicknesses, thus producing a total of two multilayer systems. The release of SB from the above-mentioned films in water was studied by means of a UV/VIS spectrophotometer at 227 nm. A first-order kinetics-type equation was used to quantitatively describe the release data. Results suggest that the film structure strongly affected the release kinetics. In fact, monolayer films showed single-stage release kinetics, whereas the two investigated multilayer systems showed two-stage release kinetics. Further, the presence of alginate beads strongly affected the SB release, thus suggesting the potential of encapsulation to control the release mechanism of active compounds.

## 1. Introduction

It is widely recognized that environmental impact generated by petroleum-derived polymers paves the route for the development of biodegradable and/or renewable alternatives. Polysaccharides, proteins and lipids are natural sources with film-forming properties that present numerous advantages such as biodegradability, edibility, biocompatibility, aesthetic appearance and, after proper fortification, barrier properties against oxygen and physical stress [1]. In this perspective, biodegradable packaging with antimicrobial properties represents a valid solution to face the quality decay of food and beverages [2,3]. The most common active packaging systems with antimicrobial properties can be made by the direct incorporation of the active agent into the polymeric material, immobilization on the polymeric surface and inclusion of a sachet/pad/filter in the package [4,5]. Numerous efforts have been made to immobilize active compounds onto the polymeric surface, by surface modification or chemical retention [6]. In this last case, both ionic and covalent immobilization require the presence of functional groups on the antimicrobial molecule and on the polymer; very often these systems need further chemical modifications to allow the active molecule being in contact with the target microorganism [7]. The binding of an agent to the surface of the package would require a molecular structure large enough to retain activity on the microbial cell wall even though bound to the plastic. Such agents are likely to be limited to enzymes or other antimicrobial proteins. Moreover, the definition of the compatibility between the polymeric matrix and active substance and the reduction in the effectiveness after melt compounding are still drawbacks to overcome [8]. The use of a sachet/pad/filter in the package also presents some drawbacks in terms of final consumer acceptance and in addition, the preservative needs to be obviously a volatile compound to be effective [9]. In this perspective, the most promising systems are the solutions that incorporate the active agent directly in the polymeric materials, being able to release it when in contact with food [10,11,12].

Most of the studies on active packaging systems relied on the preparation and utilization of films with compounds directly incorporated into the polymeric matrix and able to exert their activity when released in an uncontrolled manner from the film to the food surface [13,14]. This traditional method presents some limitations. The first one is that once the active compounds are consumed in the reaction, the protection stops, and the quality of food degrades at an increased rate. Another limitation is the inability of the active compound to selectively target the food surface where most spoilage reactions occur; consequently, an extra amount of active compound is often unnecessarily added inside the food product [6]. The capability to control releasing parameters, such as diffusivity and solubility, is particularly important to maintain the active compound concentration in a proper range to avoid either it being too low, that could be ineffective, or it being too high, that could compromise the food [15,16]. As a fact, research attention is nowadays mainly focused on controlled release systems able to preserve and modulate the active compound release over time, thus exhibiting a preserving effect toward the specific food [17,18]. Controlled release packaging can overcome the limitations of uncontrolled systems by continuously replenishing active compounds to the food surface, compensating for its consumption or degradation, so a predetermined concentration is maintained in the food to achieve the desired shelf life [19].

One of the most available methods to control the release is the adoption of multilayer systems that generally utilize the inner layer to contain the active agent, the outer layer as a barrier to prevent the loss of the active substance to the environment and a control layer to modulate the flux of release [20,21]. Emerging techniques have been used in manufacturing multilayer films with enhanced performance through surface modifications [22].

Other techniques, transferred to food packaging from both biomedical and pharmaceutical sectors to control compound release, are drug encapsulation in beads [23,24] and the adoption of nano- and microencapsulation [25,26]. Liakos et al. [27] formed calcium alginate beads encapsulating the povidone iodine complex as water disinfectants. The authors verified that controlled release of the antiseptic compound was possible when the composite beads were brought into direct contact with water or with moist media.

Alginates have been abundantly used as useful delivery systems. Various factors that impact drug release from alginate matrices, such as types of cations used in cross-linking, porosity of alginate matrices, pH effect, alginate composition, molecular weight of encapsulated drugs and modification of the functional groups in alginates, are also discussed in the literature. In addition, practical applications of the cross-linking mechanism and sol–gel transformation property of alginates are explored [28]. Among alginates, sodium alginate (NaAlg) is the most widely explored, being a non-toxic material, hydrophilic and biodegradable. The combination of NaAlg with chitosan has become quite commonplace for beads production and multilayer systems. Specifically, microspheres based on the electrostatic interaction between these two polymers have been attractive as a means to deliver many drugs because the use of organic solvents can be avoided during their preparation [29,30]. Numerous examples of entrapment for efficiency and release of active agents from chitosan/alginate complex beads are reported for medical and pharmaceutical purposes [31,32] and food applications [33,34]. The rationale for multilayer systems of chitosan and alginate is to reach a synergic effect of each biopolymer, without losing their individual intrinsic properties [35,36].

Taking into account that the release mechanisms of encapsulated agents depend on many factors such as the encapsulating material, the encapsulated substance, the geometry and morphology of the capsule, the release conditions (solvent, pH, ionic strength, temperature) and the preparation method of the capsules [37], in the current study, various process variables were considered. In particular, mono- and multilayer polymeric systems with sodium benzoate encapsulated or not in bio-based beads of alginate were realized to study the release rate of sodium benzoate from chitosan films intended for food applications.

## 2. Materials and Methods

### 2.1. Chitosan Film Preparation

High molecular weight chitosan (2 g/100 mL of distilled water) (Sigma–Aldrich, St. Louis, MO, USA) was dispersed in acetic acid (1% *v/v*) (J.T. Baker, Phillipsburg, NJ, USA) to prepare a solution with a proper concentration. Glycerol (Sigma), as plasticizer, was added to the film-forming solution at a constant concentration of 30% *w/w* of chitosan powder. The dispersion was heated (80 °C) on a hot plate for 60 min under stirring to completely dissolve chitosan. The solution was poured into a 14 cm diameter polypropylene flat dish and dried in oven at 30 °C overnight. The monolayer film was peeled from the plate and the thickness was measured using a digital micrometer (IP 65, Mitutoyo, Tokyo, Japan).

### 2.2. Sodium Alginate Film Preparation

A film-forming solution was prepared by slowly adding 4 g of sodium alginate in a constantly stirred mixture of 200 mL of distilled water with 2 g of glycerin as a plasticizer. The mixture was heated on a hot plate with stirring, until the mixture was completely dissolved. The solution was poured into 14 cm diameter polypropylene flat dishes and dried in an oven at 40 °C for about 18 h. The preformed alginate films were soaked for 5 min in CaCl_2_ solution (3 g CaCl_2_/100 mL distilled water) (Jong-Whan Rhim) and then left at room temperature to dry.

### 2.3. Active Beads Preparation

Beads were prepared by microencapsulation (Encapsulator B-395 Pro. BÜCHI, Flawil, Switzerland). To this aim, a solution of sodium alginate (0.5 g AlgNa/100 mL of distilled water) added with sodium benzoate (1.5 g BS/100 mL of distilled water) was prepared. The resulting solution was subsequently degassed to remove air bubbles using a sonicator (Ultrasonic cleaner, CP 104, CEIA, Arezzo, Italy). The solution poured into a 60 mL syringe was pumped through a nozzle of 120 µm at a rate of 9.25 mL/min and frequency of 420 Hz. The beads were collected in a solution of CaCl_2_ (3 g CaCl_2_/100 mL distilled water) under magnetic stirring.

### 2.4. Active Monolayer Films Preparation

Two monolayer active films were obtained by dispersing SB or SB-loaded alginate beads in the chitosan film-forming solution. Specifically, in the first case, 0.2 g of SB was added to the chitosan solution, whereas in the second case, 1.1 g of alginate beads was added to 55 g of chitosan solution. The first type of film was named CHT/SB, the second one CHT/ALGN-SB.

### 2.5. Multilayer Films Preparation

Multilayer active films were obtained by laying three different layers, two alginate films without any active compound with an inner layer of chitosan film containing SB alginate beads. Each layer was obtained following the procedure described above, using chitosan (4 g/100 mL of distilled water) as glue among the layers. Two multilayer active films were produced, with the two side alginate layers with two different thicknesses (120 µm for MULT-THIN and 220 µm for MULT-THIK). The two films were dried in an oven at 45 °C for two (MULT-THIN) and four days (MULT-THIK), respectively. Then, films were peeled from the plate and used for SB release.

### 2.6. Determination of Sodium Benzoate Release Kinetic

The four types of films obtained were immersed in distilled water in glass trays and shaken at 60 rpm by an orbital shaker (HS/KS 260 Basic, IKA, Germany) for three days, at 25 °C. In order to study the SB release kinetics, aliquots of water were taken at selected times that comprised very short intervals at the beginning of the assay and larger intervals after 24 h. The concentration of released SB was measured by a UV/VIS spectrophotometer (20UV, ONDA, Shangai, China) at 227 nm, using a previously performed standard curve.

### 2.7. Statistical Analysis

Fitting parameters were compared by one-way ANOVA test. A Duncan’s multiple range test, with the option of homogeneous groups (*p* < 0.05), was used to determine significance among differences within a 95% confidence interval. To this aim, STATISTICA v. 7.1 for Windows (StatSoft Inc., Tulsa, OK, USA) was used.

## 3. Results and Discussion

Even though the appropriate polymer films and information about factors controlling active compound release are available, release rates attainable with many current packaging are indeed too fast or too slow for practical use. Therefore, the rationale of this study was to model the release kinetics of SB from mono- and multilayer films to assess the influence of the bio-polymeric structure on the release rate. To this aim, different approaches were adopted: (i) dispersing SB in the simple chitosan matrix; (ii) incorporating first the drug into alginate beads and then including the active beads in the chitosan monolayer film; and finally (iii) developing multilayer structures made up of chitosan and SB alginate beads in the inner layer and alginate films with two different thicknesses as barrier and control layers.

Low-molecular weight compound diffusion in polymers is generally governed by two simultaneously occurring phenomena. A substantially stochastic phenomenon (related to Brownian motion), where the penetrant flows exclusively driven by a concentration gradient, and a relaxation phenomenon driven by the distance of the local system from the equilibrium [38]. In the case of active compound release from hydrophilic polymers into aqueous solution, such as in the case under investigation, the antimicrobial agent is initially entrapped into a hydrophilic dry polymer. When the dry film is put in contact with an aqueous solution, water molecules penetrate into the matrix [39]. The absorbed water molecules, acting as a plasticizer, increase the macromolecular mobility of the polymeric network, allowing the active compound to diffuse through the matrix into the outer water solution until a thermodynamic equilibrium between the outer solution and polymer is reached. In swelling-controlled systems, the polymeric material undergoes a transition from a glassy to a rubbery state, upon interaction with the penetrant. The polymer chains in the rubbery state, being more mobile than in the glassy state, allow the active agent to diffuse out of the matrix more rapidly. The release rate is determined by the glassy-to-rubbery transition process [12,40]. From the above description of the process, it follows that the SB release kinetics depend on the following phenomena: (1) water diffusion; (2) macromolecular matrix relaxation kinetic; and (3) diffusion of the active compound through the swollen polymeric network. Several approaches are reported in the literature to quantitatively take into account these distinct aspects of mass diffusion [40,41,42].

First-order kinetics is one possible way, most probably the simplest one, to describe the release kinetics of an antimicrobial compound entrapped in a hydrophilic polymer into a water solution:

M(t)/M_∞_ = 1 − exp(−t/a[1]) (1)

where M(t) is the amount of antimicrobial released at time t, M_∞_ is the amount of antimicrobial released at equilibrium, a[1] is the kinetic constant and t is the release time. M(t)/M∞ is dimensionless, t has the dimension of a time and a[1] has the dimension of the inverse of a time.

**Figure 1:**
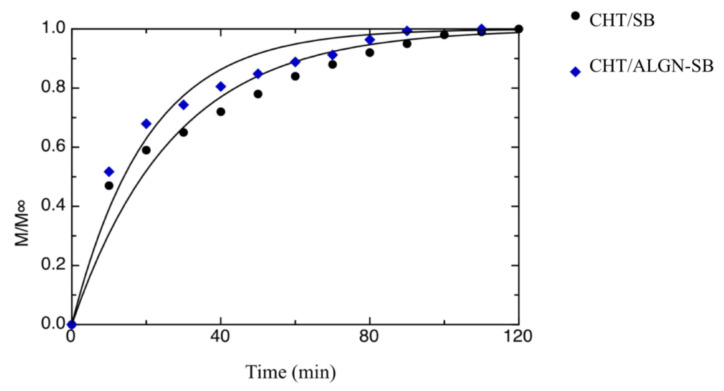
Release kinetics of sodium benzoate (SB) in water for both the CHT/SB and CHT/ALGN-SB films. The curves in the figure represent the best fitting to the data.

Figure 1 shows the release kinetics of SB in water for both CHT/SB and CHT/ALGN-SB films. The curves shown in the same figure were obtained by fitting Equation (1) to the experimental data. The kinetic constant values obtained from the fitting procedure are 27.2 ± 1.99 and 20.1 ± 1.59 min^−1^ for the CHT/SB and CHT/ALGN-SB samples, respectively. As can be inferred from the data and curves shown in the abovementioned figure, the proposed model does not perfectly fit the experimental data. This is most probably due to the fact a very simple model is used to describe the phenomena that take place during the release of a small-molecular weight compound from a glassy polymer that undergoes a glassy–rubbery transition, due to the absorption of a second low-molecular weight compound. Considering the complexity of the involved phenomena and the simplicity of the proposed model, the quality of fitting can be considered satisfying. Data show that the release rate of CHT/ALGN-SB is faster than that of the CHT/BS film. This is most probably due to the different structure of the two mentioned films. In fact, CHT/ALGN-SB is formed by alginate beads embedded into a chitosan matrix, thus due to the immiscibility between these two polymers, it is probable that chitosan does not adhere perfectly to alginate beads forming micro-channels that might create a faster pathway for SB to reach the outer media (i.e., water). A similar phenomenon has been reported by Mastromatteo et al. [18] for mono and multilayer zein-based films added with spelt bran to control the release of thymol dispersed in the bulk of the polymeric matrix. It was straightforward realizing that the release rate decreased with the increase in film thickness for both mono- and multilayer films, without the spelt bran addition. Conversely, increasing the spelt bran amount, the release rate of thymol increased in both mono- and multilayer films due to the fact that the bran addition promoted the creation of micro-channels that interconnected the thymol phase, bypassing the zein matrix, thus leading to an increase in the active agent release rate.

**Figure 2:**
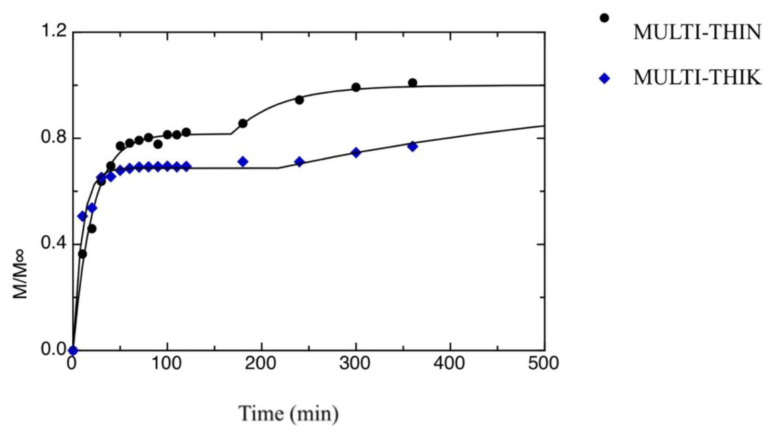
Release kinetics of SB in water from both MULT-THIN and MULT-THIK films, during the first stage of release. The curves in the figures represent the best fitting to the data.

**Figure 3:**
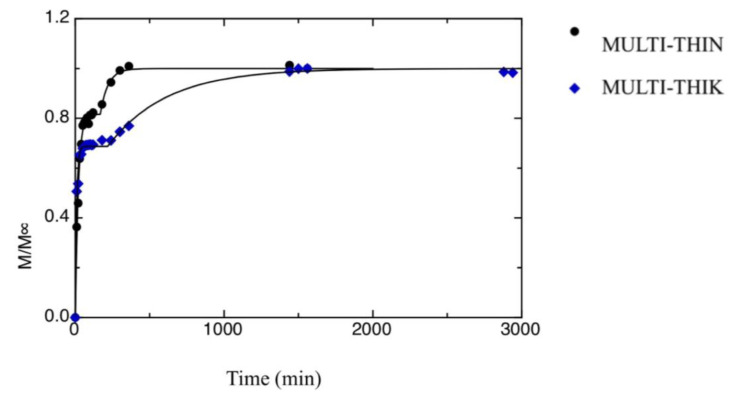
Release kinetics of SB in water from both MULT-THIN and MULT-THIK films, during the second stage of release. The curves in the figures represent the best fitting to the data.

Figure 2 and Figure 3 show the release kinetics of the two multilayer films investigated in this work, i.e., MULT-THIN and MULT-THIK. As it is evident from data shown in the figures, both samples show a two-stage release kinetics. This is due to the uneven distribution of SB between the inner and the outer layers. In fact, the multilayer films were produced according to a two-steps method, as reported in the Materials and Methods section. Initially, the antimicrobial compound is uniformly distributed in the inner chitosan film, when this latter is put in contact with the alginate film-forming solution part of SB diffused in the outer layers. Most probably, the antimicrobial compound present in the outer layers is the first to be released, whereas SB dispersed in the inner layer is released at a later stage. Han [43] summarized traditional mass transfer models and proposed mathematical approaches to describe the migration of active agents through food packaging systems consisting of single, double or triple layers. By using mass transfer models, it is possible to calculate the storage periods to maintain the active agent concentration above the critical effectiveness concentration.

In the present study, the following model is proposed to describe two-stage release kinetics:

M(t)/M_∞_ = M1(t)/M_∞_ + M2(t)/M_∞_ (2)

where M1(t) is the amount of antimicrobial released at the first stage of the release kinetics, whereas M2(t) is the amount of antimicrobial released in the second stage. M1(t) has been described according to Equation (1):

M1(t)/M_∞_ = (M1_∞_/M_∞_) [1 − exp(−t/a[1])] (3)

where M1_∞_ is the maximum amount of antimicrobial released during the first stage and a[1] is the first-stage kinetic constant. A similar equation is used to describe the antimicrobial release that takes place at the later stage:

M2(t)/M_∞_ = (1 − M1_∞_/M_∞_)[1 − exp(−(t−t0)/a[3])] (4a)

M2(t)/M_∞_ = 0 (4b)

Equation (4a) is used if t > t_0_, whereas Equation (4b) is used if t ≤ t_0_. The value a[3] is the second-stage kinetic constant, and the value t_0_ is the time at which the second release kinetics started. In fact, the proposed model has been obtained by superposing two distinct first-order kinetic models. The curves shown in Figure 2 and Figure 3 were obtained by fitting Equation (2) to the experimental data. Results from the fitting procedure are listed in Table 1. As one can infer from the data reported in Table 1, the second stage of the release process is much slower than the first one (i.e., a[1] < a[3]). This is most probably due to the fact that during the second stage, the antimicrobial molecules start from the inner chitosan layer and diffuse throughout the alginate layer to reach the outer medium. Concerning the differences between the two multilayer films, the first-stage kinetic constant of the MULT-THIN sample is bigger than that of the MULT-THIK film, whereas the contrary is true for the second-stage kinetic constant. This implies that the MULT-THIN sample has a slower release during the first stage if compared with the MULT-THIK sample and a faster release during the second stage. As reported beforehand, the release kinetics during the first stage are strongly related to the antimicrobial concentration profile throughout the multilayer film formed during the film-making. In fact, as the MULT-THIK sample has a much thicker outer alginate layer if compared with the MULT-THIN sample, it takes a longer time to dry. Therefore, it is probable that the amount of antimicrobial molecules that diffused from the inner layer and reached the outer side of the alginate layer during the multilayer film-making is bigger in the case of the MULT-THIK sample if compared with the thinner film. Concerning the second-stage kinetic constant, the MULT-THIN sample shows a value lower than the MULT-THIK film. The reason is most probably due to the fact that the antimicrobial molecules diffused throughout a thicker alginate layer to reach the outer solution in the case of the MULT-THIK sample. This is also supported by the fact that the a[5] value of the MULT-THIK sample is bigger than that of the MULT-THIN sample.

To sum up, results of this study suggest that both film structure (mono- or multilayer) and beads incorporation influenced the SB pathway to the outer water solution. Further investigation is still necessary to study the influence of both the size and formulation of alginate-based beads. From a perusal of the literature, it is found that no studies have been made on mono- or multilayer films of chitosan with sodium benzoate alginate-based beads. This study represents the first attempt in the field of food packaging. As a consequence, a direct comparison with other studies from scientific literature dealing with drug delivery through beads is not very appropriate because in most cases, beads formulation consists of alginates blended with other polymeric materials to enhance the entrapment efficacy [44,45], or cross-linked with chitosan [46]. Similarly, examples of chitosan films incorporating generally recognized as safe (GRAS) antimicrobials as sodium benzoate are found in the scientific literature; however, these studies are not focused on the kinetic release of SB but rather on exclusively assessing the effect of active chitosan films on target microorganisms in model systems or real food [47,48,49].

## 4. Conclusions

The release kinetics of SB from polymeric films affected by the film structure were addressed in this study. In particular, four different films were investigated, two monolayer films (i.e., SB uniformly distributed in a monolayer chitosan film, SB-loaded beads uniformly dispersed into a chitosan monolayer film) and two multilayer films, that were obtained by laying two alginate films on an inner chitosan film in which SB-loaded beads were uniformly dispersed. A first-order kinetics-type equation was used to quantitatively describe the release data. Results suggest that the release kinetics are strongly influenced by the film structure and by the procedure used to produce the film. In fact, monolayer films show single-stage release kinetics, whereas the two investigated multilayer films show two-stage release kinetics. Further, the presence of alginate beads can strongly affect the SB release kinetics as it may induce a preferential pathway of diffusing molecules to reach the outer water solution.

## Figures and Tables

**Table 1:** Fitting parameters of multilayer films.

	a[1] [min^−1^]	a[3] [min^−1^]	M1_∞_/M_∞_	t_0_ [min]
MULT-THIN	20.3 ± 1.03 ^a^	52.6 ± 18.4 ^b^	0.816 ± 9.62 × 10^−3 a^	168 ± 10.3 ^b^
MULT-THIK	9.32 ± 0.757 ^b^	393 ± 110 ^a^	0.687 ± 7.51 × 10^−3 b^	219 ± 30.3 ^a^

Data in the same column with different letters are significantly different (*p* < 0.05).
